# Seroprevalence and coprological prevalence of liver fluke *Fasciola hepatica* in cattle and sheep from Santander department, Colombia

**DOI:** 10.1590/S1984-29612023071

**Published:** 2023-12-04

**Authors:** Nelson Uribe Delgado, Andrés Esteban Pereira, Ruth Aralí Martínez, Angel Alberto Florez Muñoz, Juan Carlos Pinilla

**Affiliations:** 1 Escuela de Microbiología, Universidad Industrial de Santander, Bucaramanga, Colombia; 2 Departamento de Ciencia Animal, Facultad de Veterinaria, Universidad Concepción, Chillán, Chile; 3 Facultad de Ciencias Agrícolas y Veterinarias, Universidad de Santander, Bucaramanga, Colombia

**Keywords:** Cattle, Colombia, fasciolosis, livestock, sheep, Bovinos, Colômbia, fasciolose, pecuária, ovinos

## Abstract

*Fasciola hepatica* is a parasite with a worldwide distribution that affects several mammals, including humans, and is considered a public health problem. Therefore, the aim of this study was to determine the prevalence of *Fasciola hepatica* in humans, cattle and sheep, as well as to evaluate factors associated with the prevalence. A total of 185 serum samples from sheep, 290 from cattle, and 114 from humans were collected and processed using an in-house developed ELISA to detect IgG antibodies against *F. hepatica*. Additionally, 185 stool samples from sheep and 290 from cattle were examined using a Dennis sedimentation technique. Risk factors were analyzed using epidemiological surveys. The overall seroprevalence was 46.5% (86/185) in sheep, 32.5% (94/289) in cattle, and no humans tested positive for the infection. The coprological prevalence was 47.7% (86/180) in sheep and 33.7% (98/290) in cattle. Female gender and cattle living with alternate grazing management showed 2.5 and 6.5 times higher probability of infection, respectively. Bovines coexisting with sheep exhibited a higher risk of infection (odds ratio [OR]=4.3) compared to those without sheep. We concluded that *F. hepatica* in cattle and sheep has an endemic behavior, and therefore represents a problem of public health for rural communities.

## Resumo

*Fasciola hepatica* é um parasita com distribuição mundial que afeta diversos mamíferos, inclusive humanos, sendo considerado um problema de saúde pública. Portanto, o objetivo deste estudo foi determinar a prevalência de *Fasciola hepatica* em humanos, bovinos e ovinos, bem como avaliar fatores associados à prevalência de infecção em bovinos. Um total de 185 amostras de soro de ovinos, 290 de bovinos e 114 de humanos foram coletadas e processadas, usando-se ELISA desenvolvido internamente para detectar anticorpos IgG contra *F. hepatica*. Além disso, 185 amostras de fezes de ovinos e 290 de bovinos foram examinadas, usando-se uma técnica de sedimentação modificada de Dennis. Os fatores de risco foram analisados ​​por meio de inquéritos epidemiológicos. A soroprevalência de *F. hepatica* foi de 46,5% (86/185) em ovinos, 32,5% (94/289) em bovinos, e nenhum humano apresentou resultado positivo para a infecção. A prevalência coprológica foi de 47,7% (86/180) em ovinos e 33,7% (98/290) em bovinos. Fêmeas e gado vivendo com pastejo alternado, apresentaram probabilidade 2,5 e 6,5 vezes maior de infecção, respectivamente. Bovinos coexistindo com ovelhas apresentaram maior probabilidade de infecção (*odds ratio* [OR]=4,3) em comparação com aqueles sem ovelhas. Concluí-se que a *F. hepatica* em bovinos e ovinos tem comportamento endêmico e, portanto, representa um problema de saúde pública para as comunidades rurais.

## Introduction

Liver fluke, *Fasciola hepatica*, is a parasitic helminth of the trematode class that causes a chronic disease affecting the liver and bile ducts in ruminants, as well as various other mammals such as sheep, goats, horses, deer, and humans. The primary etiological agent responsible for fasciolosis in Colombia, as well as in other countries in America, Africa, and Europe, is predominantly *F. hepatica* (González et al., 2011; Mas-Coma, 2005; Rokni et al., 2002). Infection in ruminants and other definitive hosts occurs through oral ingestion of water, pasture, and food contaminated with metacercariae (Cordero & Rojas, 1999). This trematode parasite causes substantial economic losses in animal production and poses a threat to food security, with its adult stage relying on Lymnaeidae snails to complete its biological cycle.

Fasciolosis is acknowledged as an emerging and re-emerging zoonotic disease. Numerous reports indicate that in countries such as Argentina, Bolivia, Peru, Uruguay, Brazil, and Chile, the prevalence of animal fasciolosis averages around 57% (Carmona & Tort, 2017). Globally, an estimated 2.4 to 17 million people are infected, while 180 million individuals are at risk of acquiring the infection (Mehmood et al., 2017). Although there have been few reported cases of human fasciolosis in Colombia, various studies conducted in the country provide important insights into the prevalence of animal fasciolosis. Historically, the coprological prevalence of *F. hepatica* in Colombia is 25% (Estrada Orrego et al., 2006), however, several authors have reported seroprevalence rates of 39.4% to 40% in cattle from the Cundinamarca department and Bogota DF (Giraldo Forero et al., 2016). Similarly, Sierra et al. (2018) reported an 82% prevalence rate in sheep from the Cesar department. In the Quindío department, a prevalence of 3.7% was found in cattle (Recalde-Reyes et al., 2014), while a study conducted in 2013 in Pamplona, Norte de Santander, showed a prevalence rate of 93.7% (Palma et al., 2014).

The García Rovira region and Onzaga municipality, located in the Northeastern part of Colombia, are significant agricultural regions characterized by small farms dedicated to cattle and sheep production, as well as vegetable cultivation for human consumption. Therefore, studying the prevalence of fasciolosis in these regions of Santander can contribute to a better understanding of the disease in endemic areas of Colombia. Consequently, the primary objective of this research was to determine the seroprevalence of *Fasciola hepatica* in humans, cattle, and sheep in the Garcia Rovira region and Onzaga municipality of the Santander department, as well as to evaluate factors associated with the prevalence of infestation in livestock.

## Materials and Methods

### Study region and sampling design

**Figure 1:**
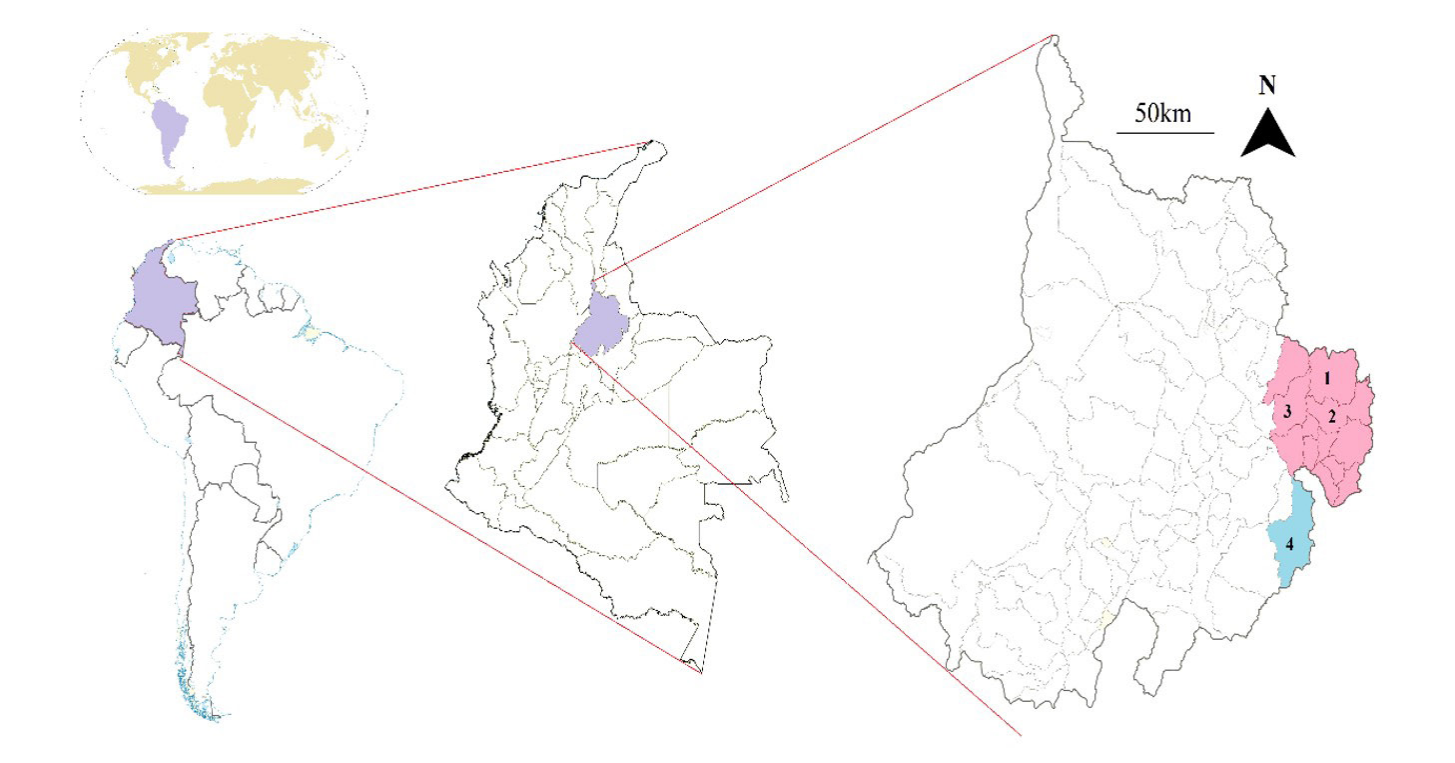
Location of García Rovira region (pink) municipalities: 1-Cerrito, 2-Concepción, 3-San Andrés and 4- Onzaga municipality (light blue) in Santander department, Colombia.

The research was conducted in the Garcia Rovira region, specifically in the municipalities of Cerrito, Concepción, and San Andrés, as well as the Onzaga municipality in the Santander department of Colombia (Gobernación de Santander, 2017). The geographic coordinates of the study area were 6°47'58.5”N - 72°31'59.1”W for the Garcia Rovira region and 6°96'53.1”N - 72°75'73.3”W for the Onzaga municipality (Figure 1). The Garcia Rovira region is located approximately 100 km from the Venezuelan border and is characterized by mountainous terrain with complex slopes ranging from 25% to 50%. The region has a humid mountain forest within a cold thermal floor known as “paramo,” situated at an elevation between 3000 and 4000 meters above sea level. The mean annual temperature ranges from 6°C to 12°C, and the mean annual rainfall ranges from 2000 to 4000 mm. The Onzaga municipality shares similar geographic characteristics with the Garcia Rovira region (Gobernación de Santander, 2017).

A descriptive and transversal study design was employed. A total of 61 small-scale livestock farms, with an average area of 32 hectares, were visited between September 2018 and April 2019. These farms predominantly raised mixed cattle and sheep and engaged in various agricultural activities. To determine the sample size, the formula for known populations (Thrusfield, 2007) was used, considering an expected prevalence of 15%, a margin of error of 5%, and a 95% confidence interval. The resulting sample size (“n”) was calculated to be 475 animals. The number of farms selected was proportional to the livestock population of each municipality, and the sample size within each farm and for different age groups within each farm was determined proportionally. Consequently, each farm provided between 7 to 10 samples, with an average of 8.6 samples per farm. This sampling strategy yielded a total of 290 cattle and 185 sheep samples. Blood and stool samples were collected from each animal, while 114 human serum samples were obtained from volunteers permanently residing on the farms and having a probability of *F. hepatica i*nfection risk.

### Sample collection and laboratory analysis

Weekly fecal samples were collected from each sheep and cattle by collecting approximately 5 to 10 g of feces from the rectum. The samples were placed in previously labeled sterile polyethylene bags and preserved with two drops of 10% formaldehyde. The fecal samples were transported to the laboratory within 8 hours for processing. A coprological technique described by Correa et al. (2016) was employed for fecal sample analysis.

For each animal, 5 mL of whole blood was collected aseptically in a sterile tube without EDTA (ethylenediaminetetraacetic acid) using a disposable syringe from the coccygeal vein. The serum was obtained by centrifugation at 3600 g for 10 minutes and stored at -20°C until further analysis. Serum samples were analyzed for specific anti-*F. hepatica* antibodies using an in-house enzyme-linked immunosorbent assay (ELISA) with 100% sensitivity in humans, sheep, and cattle, and specificities of 97%, 85.2%, and 96.2%, respectively (Sierra Balcárcel et al., 2017). The results were read in a microplate photometer, measuring the optical density (OD) at 450 nm. A cut-off value of 0.15 for humans, 0.18 for sheep, and 0.28 for cattle was set to determine positive results, and the results were expressed as percent positive (PP). A farm was considered positive if at least one cattle or sheep tested positive by any technique.

### Statistical analysis

The seroprevalence and coprological prevalence results were analyzed using the Chi-square test (X^2^) to determine the association between independent variables and prevalence values. Variables showing statistical significance at a 5% level were included in a multivariable logistic regression analysis (Aguayo Canela, 2007).

## Results

A total of 474 animals were screened from 61 farms located in the Garcia Rovira region and Onzaga municipality. The overall seroprevalence for liver fluke *F. hepatica* in cattle and sheep from the region under study was 37.9% (180/474, CI95% 33.5-46.2). Table 1 presents the seroprevalence values in cattle and sheep with different potential risk factors. The serological prevalence in cattle was 32.5% (94/289, 95%CI 27.4% - 38.1%), while in sheep, it was 46.5% (86/185, 95%CI 40.1% - 54.5%). No human sera were positive for *F. hepatica* infection. Chi-square tests revealed a statistical association (X^2^ = 46.1, p<0.05) between seroprevalence values in cattle and the four municipalities: 51.2% (21/41) in Concepcion, 6.3% (6/96) in San Andres, 46.2% (24/52) in Cerrito, and 43% (43/100) in Onzaga municipality. The highest seroprevalence was observed in Concepcion municipality. Age category analysis showed that the youngest cattle (<12 months) had the highest seropositivity (44.4%), although there was no statistical significance (p>0.05) regarding age. Among the other variables in cattle, *F. hepatica* showed statistically significant associations (p<0.05) with sex, pasture management, water source, and animal mixture. Seroprevalence was significantly higher in females (34.9%), in alternated grazing management (32.9%), when cattle drank water from the river (41.2%), and when cattle were mixed with sheep (63.3%).

**Table 1:** Seroprevalence values of anti-*Fasciola hepatica* antibodies in cattle and sheep with different potential risk factors in the Santander department, Colombia (univariate analysis).

**Factor**	**Categories**	**N°**	**Positives**	**SP (%)**	**X^2^**	***P-value***
Cattle						
Garcia Rovira region	Concepcion	41	21	51.2		
	San Andres	96	6	6.3		
	Cerrito	52	24	46.2		
Onzaga municipality	Onzaga	100	43	43	46.1	0.000
Age	<12	36	16	44.4		
	12-24	18	5	27.8		
	>24	235	73	31.1	2.7	0.25
Sex	Male	44	8	18.2		
	Female	246	86	34.9	4.86	0.027
Pasture management	Alternated	146	48	32.9		
	Rotative	43	3	7	11.3	0.001
Water source/animal consumption	River	136	56	41.2		
	Spring water	102	22	21.6		
	Aqueduct	51	16	31.4	10.2	0.006
Feces management	No	231	72	31.2		
	Yes	58	22	37.9	0.96	0.32
Animal mixture	No	259	75	29		
	Yes	30	19	63.3	14.5	0.000
Overall		289	94	32.5		
Sheep						
Garcia Rovira region	Concepcion	70	20	28.6		
	San Andres	21	0	0		
	Cerrito	94	66	70.2	48.5	0.000
Onzaga municipality	Onzaga	-	-	-		-
Age	<12	25	10	40		
	12-24	48	16	33.3		
	>24	112	60	53.6	6.02	0.049
Sex	Male	42	11	26.2		
	Female	143	7	52.4	8.99	0.003
Pasture management	Alternated	157	78	49.7		
	Rotative	28	8	28.6	4.25	0.039
Water source/animal consumption	River	37	12	32.4		
	Spring water	131	69	52.7		
	Aqueduct	17	5	29.4	6.93	0.031
Feces management	No	119	54	45.4		
	Yes	66	32	48.5	0.16	0.68
Animal mixture	No	118	48	40.7		
	Yes	67	38	56.7	4.42	0.036
Overall		185	86	46.5		

N◦: number of samples; SP (%): percentage of seroprevalence; X^2^: chi square value; Statistically significant (*P < .05*).

In sheep, statistically significant associations were found (X^2^ = 48.5, p<0.05) between seroprevalence results and municipalities: 28.6% (20/70) in Concepcion, 0% (0/21) in San Andres, and 70.2% (66/94) in Cerrito (Table 1). The highest seroprevalence was observed in Cerrito municipality. Regarding age and sex, different percentages of parasitism were observed in sheep, suggesting statistical significance (p<0.05). *F. hepatica* also showed statistically significant associations (p<0.05) with alternated grazing management, sheep drinking water from the spring, and sheep mixed with cattle. However, there was no statistical association (p>0.05) between seroprevalence and feces management in sheep (Table 1).

The percentage of positive fecal samples in cattle was 33.7% (98/290, 95%CI 28.9% – 39.7%), and in sheep, it was 47.7% (86/180, 95%CI 40.1% – 54.5%). The coprological prevalence was higher (p<0.05) in cattle from Onzaga municipality (47.5%, 95%CI 37.9% - 57.2%) than those from the Garcia Rovira region (27%, 95%CI 21.2% - 33.7%). Of the total number of examined animals, 86 sheep were positive by both serology and coprology, while 77 cattle were positive in both tests.

Regarding risk factors in catle (Table 2), female showed 2.5 (OR = 2.5, CI95% = 1.08-5.4) times higher risk of infection than male. Pasture management alternated and animal mixture (yes) showed 6.5 (OR = 6.5, CI95% = 1.9-22.1) and 4.3 (OR = 4.3, CI95% = 1.9-9.3) times higher probability for infection with liver fluke, respectively. With respect to sheep (Table 3), female and animals from Cerrito municipality showed 2.3 (OR=2.3, CI95% = 0.9-5.8) and 10.5 (OR = 10.5, CI95% = 3.9-28.1) times higher probability for infection, respectively.

**Table 2:** Results of a multivariable regression logistic analysis for *Fasciola hepatica* infections in cattle in the Santander department, Colombia.

**Factor**	**Categories**	**B**	**E.T**	***P*-value**	**Exp (β)**	**CI (95%)**
Onzaga municipality	Onzaga	-	-	-	1	-
Garcia Rovira region	Concepcion	0.33	0.37	0.03*	1.4	0.6-2.9
	San Andres	-2.4	0.47	0.3	0.08	0.03-0.2
	Cerrito	0.1	0.34	0.7	1.1	0.6-2.3
Sex	Male	-	-	-	1	-
	Female	0.89	0.41	0.03*	2.5	1.08-5.4
Pasture management	Rotative	-	-	-	1	-
	Alternated	1.87	0.62	0.003*	6.5	1.9-22.1
Watersource/animal consumption	Aqueduct	-	-	-	1	-
	River	0.42	0.34	0.22	1.5	0.7-3.03
	Spring water	-0.5	0.38	0.18	0.6	0.3-1.28
Animal mixture	No	-	-	-	1	-
	Yes	1.44	0.4	0.000*	4.3	1.9-9.3

B: estimated value B; E.T: standard error; 1: reference category; Exp(β): OR; CI: confidence interval; Statistically significant (*P* < .05).

* Risk factor.

**Table 3:** Results of a multivariable regression logistic analysis for *Fasciola hepatica* infections in sheep in the Santander department, Colombia.

**Risk factor**	**Categories**	**B**	**E.T**	***P*-value**	**Exp (β)**	**CI (95%)**
Garcia Rovira region	Concepcion	-	-	-	1	-
	San Andres	-20.4	8610	0.99	0	0
	Cerrito	2.3	0.5	0.000*	10.5	3.9-28.1
Age	<12	-	-	-	1	-
	12-24	-1.02	0.64	0.11	0.36	0.1-1.3
	>24	-0.07	0.56	0.9	0.9	0.3-2.8
Sex	Male	-	-	-	1	-
	Female	0.87	0.45	0.049*	2.3	0.9-5.8
Pasture management	Rotative	-	-	-	1	-
	Alternated	0.82	0.22	0.18	2.2	0.6-7.7
Watersource/animal consumption	Aqueduct	-	-	-	1	
	River	-0.18	0.72	0.8	0.8	0.2-3.4
	Spring water	-1.3	0.96	0.17	0.27	0.04-1.8
Animal mixture	No	-	-	-	1	-
	Yes	-0.76	0.47	0.1	0.46	0.18-1.1

B: estimated value B; E.T: standard error; 1: reference category; Exp(β): OR; CI: confidence interval; Statistically significant (*P* < .05).

* Risk factor.

## Discussion

Liver fluke *F. hepatica* is a helminth parasite presents around the world which causes a chronic disease that affect the liver and bile ducts on ruminants and a variety of mammals including humans (Ichikawa-Seki et al., 2017). Therefore, the aim of this research was to investigate the seroprevalence of *F. hepatica* in ruminants and humans from the Garcia Rovira region and Onzaga municipality, department of Santander, Colombia.

The overall seroprevalence in study area was 37.9%, being 46.5% in sheep and 32.5% in cattle. In García Rovira region, the seroprevalence was 26.9% in cattle and 46.5% in sheep, while the seroprevalence in cattle from the Onzaga municipality was 43%. These results agree with those reported by (Giraldo Forero et al., 2016; Chaparro et al., 2016; Pinilla et al., 2019, 2020a, b) who reported similar seroprevalence results in ruminants raised in Colombian farms located > 2000 masl. Equally, the results obtained were similar to those of some other studies conducted in Peru and Venezuela, which described similar results in cattle farms located > 2000 masl (Ticona et al., 2010; Gauta et al., 2011). Nevertheless, the results obtained differ to those informed by other authors, who reported lower prevalence results in cattle from Venezuela and Colombia (Angulo-Cubillán et al., 2013; Recalde-Reyes et al., 2014; Pinilla et al., 2018), and sheep from Boyaca, Colombia (Pulido-Medellin et al., 2014).

The region under study shows optimal climatological conditions for the viability of Lymnaeidae snails and develop of the infection by *F. hepatica* for the animals. Therefore, grazing animals favor the presence of the trematode, due the animals are exposed to the infectious stages (Valderrama, 2016). According to Pereira et al. (2020) the Lymnaeidae snails has been reported in these areas of Colombia, since these areas have crystalline water and aquatic plants as watercress (*Nasturtium officinale*) necessary for the transmission of the parasite (Giraldo Pinzpón & Álvarez Mejía, 2013). Therefore, these plants serve as source of infection of metacercariae, which it perpetuates the parasitic infection in the farms (Mas-Coma et al., 2001; Giraldo-Pinzón et al., 2016). Mas-Coma et al. (2001) indicated that *F. hepatica* has been informed in farms located above 2000 masl, where there is a humid forest climate with the water temperatures around 10° C. Therefore, the presence of *F. hepatica* in ruminants depend mainly in factors like low temperature and long periods of rain necessary for the presence of snails and the circulation of liver fluke among agricultural communities, representing a food safety problem. Despite there are no reports on livers confiscation in the study region, the high seroprevalence results of liver fluke *F. hepatica* found in ruminants could be a big reason to performed on public health research of the trematode in the region.

Regarding risk factors in cattle, female showed higher probability for infection than male (OR= 2.5) (Table 2). Probably, situations of stress in cows due to heat, calving, lactating and weaning cause immunosuppression and increased parasitic infection rate (Odeón & Romera, 2017). Cattle living with an alternated grazing management showed 6.5 times higher risk infection with *F. hepatica* than animals grazing in rotative pasture, due alternative grazing consists of changing animals from different pastures. On the other hand, rotative grazing consists of dividing a paddock into two parts of similar dimensions, so that the animals graze on one part of the paddock, while the other remains at rest. This management can limit the contamination of the pasture and can be an option to use strategic treatments to reduce the levels of infection by snails (Knubben-Schweizer & Torgerson, 2015). Cattle grazing pasture with sheep showed higher probability of risk than when were alone. Although sheep showed higher prevalence (46.5%) than cattle (32.5%) in this study, the mixture of both species can act as a risk factor, due sheep are more susceptible to be infected by liver fluke *F. hepatica*, since this animal species do not develop resistance against new infections, and therefore contributes permanently to disseminate the infections for a long time (Olaechea, 2007).

Even though people in García Rovira region and Onzaga municipality lives in endemic animal fasciolosis areas, human infection with *F. hepatica* was no found in this study area. However, during the sampling it was evident that the water source for people was not the same as for the animals, and in most cases the families took the water from some source of water near the farm, where the animals had no access. On the other hand, snails were not found in watercress and other vegetables, but only in the water sources where the animals consumed. Despite open-air water irrigation channels to supply homes are considered the primary source of infection for human fascioliasis in endemic areas (Marcos et al., 2005), the area studied had no water circulation system that avoid mollusk development, and this could be a reason for the low prevalence rate of snails in the study area. The common water source for human and animal consumption does not necessarily imply that there is the same risk for humans and livestock. Water can have the same origin (aqueduct, cistern, spring), but the cycle is only completed when that water is established in places where snails can develop. Similarly, this water must be susceptible to contamination with fecal matter from infected animals. Therefore, although the water source can be the same, the cycle is only completed in the irrigation canals, drinking fountains, ponds, wells and other places where the consumers are animals.

## Conclusion

The presence of antibodies anti- *F. hepatica* (37.9%) in cattle and sheep of the region under study is confirmed, which suggests an endemic behavior of this parasitosis, and therefore represents a problem of food security and public health for rural communities.
